# On Fuzzy Ideals of *BL*-Algebras

**DOI:** 10.1155/2014/757382

**Published:** 2014-04-17

**Authors:** Biao Long Meng, Xiao Long Xin

**Affiliations:** ^1^Department of Mathematics, Northwest University, Xi'an 710127, China; ^2^College of Science, Xi'an University of Science and Technology, Xi'an 710054, China

## Abstract

In this paper we investigate further properties of fuzzy ideals of a *BL*-algebra. The notions of fuzzy prime ideals, fuzzy irreducible ideals, and fuzzy Gödel ideals of a *BL*-algebra are introduced and their several properties are investigated. We give a procedure to generate a fuzzy ideal by a fuzzy set. We prove that every fuzzy irreducible ideal is a fuzzy prime ideal but a fuzzy prime ideal may not be a fuzzy irreducible ideal and prove that a fuzzy prime ideal **ω** is a fuzzy irreducible ideal if and only if *ω*(0) = 1 and |Im*⁡*(*ω*)| = 2. We give the Krull-Stone representation theorem of fuzzy ideals in *BL*-algebras. Furthermore, we prove that the lattice of all fuzzy ideals of a *BL*-algebra is a complete distributive lattice. Finally, it is proved that every fuzzy Boolean ideal is a fuzzy Gödel ideal, but the converse implication is not true.

## 1. Introduction

It is well-known that an important task of the artificial intelligence is to make computer simulate human being in dealing with certainty and uncertainty in information. Logic gives a technique for laying the foundations of this task. Information processing dealing with certain information is based on the classical logic. Nonclassical logic includes many valued logic and fuzzy logic which takes the advantage of the classical logic to handle information with various facets of uncertainty [1], such as fuzziness and randomness. Therefore, nonclassical logic has become a formal and useful tool for computer science to deal with fuzzy information and uncertain information. Fuzziness and incomparability are two kinds of uncertainties often associated with human's intelligent activities in the real word, and they exist not only in the processed object itself, but also in the course of the object being dealt with.

The notion of BL-algebra was initiated by Hájek [2] in order to provide an algebraic proof of the completeness theorem of Basic Logic (*BL*, in short). A well known example of a *BL*-algebra is the interval [0,1] endowed with the structure induced by a continuous *t*-norm. *MV*-algebras [3], Gödel algebras, and Product algebras are the most known class of *BL*-algebras. Cignoli et al. [4] proved that Hájek's logic really is the logic of continuous *t*-norms as conjectured by Hájek. Filters theory plays an important role in studying *BL*-algebras. From logic point of view, various filters correspond to various sets of provable formulae. Hájek introduced the notions of filters and prime filters in *BL*-algebras and proved the completeness of Basic Logic using prime filters. Turunnen [5–7] studied some properties of deductive systems and prime deductive systems. Haveshki et al. [8, 9] introduced (positive, fantastic) implicative filters in *BL*-algebras and studied their properties.

The concept of fuzzy sets was introduced by Zadeh [10]. At present, these ideals have been applied to other algebraic structures such as groups and rings. Liu et al. ([11, 12]) introduced the notions of fuzzy filters and fuzzy prime filters in *BL*-algebras and investigated some of their properties. Zhan et al. [13–16] introduced some kinds of generalized fuzzy filters in *BL*-algebras and described their relations with ordinary fuzzy filters. Another important notion of *BL*-algebras is ideal, which was introduced by Hájek [2]. Some properties of ideals were investigated by Saeid [17]. Fuzzy ideal theory in *BL*-algebras is studied by Zhang et al. [15]. The notions of fuzzy prime ideals and fuzzy Boolean ideals are introduced.

In the present paper we will systematically investigate fuzzy ideal theory of *BL*-algebras. The paper is organized as follows. In Section 2, we recall some basic definitions and results of *BL*-algebras. In Section 3, we provide a procedure to generate a fuzzy ideal by a fuzzy set. In Section 4, the notions of fuzzy irreducible ideals and fuzzy Gödel ideals are introduced. We give a new definition of fuzzy prime ideals in a *BL*-algebra and prove that it is equivalent to one in Zhang et al. [15]. We prove that every fuzzy irreducible ideal in a *BL*-algebra is a fuzzy prime ideal and give an example to show that a fuzzy prime ideal may not be a fuzzy irreducible ideal; also we prove that a fuzzy prime ideal *ω* is a fuzzy irreducible ideal if and only if *ω*(0) = 1 and |*Im*⁡(*ω*)| = 2. Furthermore, we give the Krull-Stone representation theorem of fuzzy ideals in *BL*-algebras. In Section 5, we prove that the lattice of all fuzzy ideals of a *BL*-algebra is a complete distributive lattice. Finally, in Section 6, we introduce the notion of fuzzy Gödel ideals and investigate basic properties of fuzzy Gödel ideals and prove that every fuzzy Boolean ideal is a fuzzy Gödel ideal but the converse implication is not true.

## 2. Preliminaries

Let us recall some definitions and results on *BL*-algebras.

**Definition 1 (see [2])**

An algebra (*A*; ∧, ∨, ∗, →, 0,1) of type (2,2, 2,2, 0,0) is called a *BL*-algebra if it satisfies the following conditions.

(BL1) (*A*;  ∧, ∨, 0,1) is a bounded lattice.
(BL2) (*A*;  ∗, 1) is a commutative monoid.
(BL3) *x*∗*z* ≤ *y* if and only if *z* ≤ *x* → *y* (residuation).
(BL4) *x*∧*y* = *x*∗(*x* → *y*); thus *x*∗(*x* → *y*) = *y*∗(*y* → *x*) (divisibility).
(BL5) (*x* → *y*)∨(*y* → *x*) = 1 (prelinearity).

Throughout this paper, let *A* denote a *BL*-algebra.

**Proposition 2 (see [5, 9])**

Let *A* be a *BL*-algebra. For all *x*, *y*, *z* ∈ *A*, the following are valid:

1. *x*∗(*x* → *y*) ≤ *y*,
2. *x* ≤ *y* → (*x*∗*y*),
3. *x* ≤ *y if and only if x* → *y* = 1,
4. *x* → (*y* → *z*) = (*x*∗*y*) → *z* = *y* → (*x* → *z*),
5. *x* ≤ *y implies z* → *x* ≤ *z* → *y*, *y* → *z* ≤ *x* → *z*,
6. *y* ≤ (*y* → *x*) → *x*,
7. (*x* → *y*)∗(*y* → *z*) ≤ *x* → *z*,
8. *y* → *x* ≤ (*z* → *y*)→(*z* → *x*),
9. *x* → *y* ≤ (*y* → *z*)→(*x* → *z*),
10. *x*∨*y* = [(*x* → *y*) → *y*]∧[(*y* → *x*) → *x*],
11. *x* ≤ *y implies y* ^−^ ≤ *x* ^−^,
12. 1 → *x* = *x*,*x* → *x* = 1,*x* → 1 = 1,
13. *x* ≤ *y* → *x, or equivalently, x* → (*y* → *x*) = 1,
14. ((*x* → *y*) → *y*) → *y* = *x* → *y*,
15. 1^−^ = 0, 0^−^ = 1,
16. 1^−−^ = 1, 0^−−^ = 0*; that is, *0 and 1* are involutions,*
17. (*x*∨*y*)^−^ = *x* ^−^∧*y* ^−^, (*x*∧*y*)^−^ = *x* ^−^∨*y* ^−^,
18. *x* → *y* ≤ *x*∗*z* → *y*∗*z*,
19. *x* → *y* ≤ *x*∧*z* → *y*∧*z*,
20. *x* → *y* ≤ *x*∨*z* → *y*∨*z*,

*where x* ^−^ = *x* → 0.

The set of all natural numbers is denoted by *N*. We denote

(1) $$\begin{array}{c} x^{0}=1,x^{1}=x,x^{2}=x*x,\ldots ,x^{n}=\underset{n}{\underset{︸}{x*\cdots *x}}. \end{array}$$

A *BL*-algebra *A* is a Gödel algebra if *x* ^2^ = *x* for any *x* ∈ *A*. An element *x* is involutory, if *x* ^−−^ = *x*.

In this paper we will often use the identity (*y* → *x* ^−^)^−−^ = *y* → *x* ^−^ for any *x*, *y* ∈ *A* (see [18]).

**Definition 3 (see [2])**

A nonempty subset *I* of *BL*-algebra *A* is called an ideal of *A* if it satisfies:

(I1) 0 ∈ *I*,
(I2) *x* ∈ *I* and (*x* ^−^ → *y* ^−^)^−^ ∈ *I* implies *y* ∈ *I* for all *x*, *y* ∈ *A*.

A proper ideal *I* of *BL*-algebra *A* is called a prime ideal of *A* if *x*∧*y* ∈ *I* implies *x* ∈ *I* or *y* ∈ *I* for all *x*, *y* ∈ *A*.

**Lemma 4 (see [18])**

Let *I* be an ideal in *A* and *a* ∈ *A* − *I*. Then there is a prime ideal *P* of *A* such that *I*⊆*P* and *a* ∉ *P*.

**Definition 5 (see [18])**

Let *I* be an ideal of *BL*-algebra *A*. *I* is called a Gödel ideal if it satisfies (*x* ^−^ → (*x* ^−^)^2^)^−^ ∈ *I* for any *x* ∈ *A*.

A fuzzy set in *A* is a mapping *μ* : *A* → [0,1]. Let *μ* be a fuzzy set in *A* and *t* ∈ [0,1], the set *μ*_*t*_ = {*x* ∈ *A* | *μ*(*x*) ≥ *t*} is called a level subset of *μ*.

The notations 1_*A*_ and 0_*A*_ represent two special fuzzy sets in *A* satisfying 1_*A*_(*x*) = 1 for any *x* ∈ *A* and 0_*A*_(*x*) = 0 for any *x* ∈ *A*, respectively.

For any fuzzy sets *μ*, *ν*, *μ*_*λ*_ (*λ* ∈ Λ) in *A* where Λ is an index set, we define *μ*∨*ν*, *μ*∧*ν*, ∨{*μ*_*λ*_ : *λ* ∈ Λ} and ∧{*μ*_*λ*_ : *λ* ∈ Λ} as follows: for all *x* ∈ *A*,

(2) $$\begin{array}{c} \left(\mu \vee \nu \right)\left(x\right)=\mu \left(x\right)\vee \nu \left(x\right),\\ \left(\mu \wedge \nu \right)\left(x\right)=\mu \left(x\right)\wedge \nu \left(x\right),\\ \vee \left\{\mu_{\lambda }:\lambda \in \Lambda \right\}\left(x\right)=\vee \left\{\mu_{\lambda }\left(x\right):\lambda \in \Lambda \right\},\\ \wedge \left\{\mu_{\lambda }:\lambda \in \Lambda \right\}\left(x\right)=\wedge \left\{\mu_{\lambda }\left(x\right):\lambda \in \Lambda \right\}. \end{array}$$

By *μ* ≤ *ν* we mean that *μ*(*x*) ≤ *ν*(*x*) for all *x* ∈ *A*.

**Definition 6 (see [15])**

Let *A* be a *BL*-algebra. A fuzzy set *μ* in *A* is called a fuzzy ideal of *A* if, for all *x*, *y* ∈ *A*,

(FI1) *μ*(0) ≥ *μ*(*x*),
(FI2) *μ*(*y*) ≥ *μ*(*x*)∧*μ*((*x* ^−^ → *y* ^−^)^−^).

**Proposition 7 (see [15])**

Let *μ* be a fuzzy set in *A*. Then *μ* is a fuzzy ideal if and only if, for each *t* ∈ [0,1], *μ*_*t*_ is either empty or an ideal of *A*.

## 3. Fuzzy Ideal Generated by a Fuzzy Set

In this section, we give a procedure to construct the fuzzy ideal generated by a fuzzy set. First of all we give some further properties of fuzzy ideals in *BL*-algebras. The set of all fuzzy sets in *BL*-algebra *A* is denoted by *F*(*A*) and the set of all fuzzy ideals in *A* is denoted by FI(*A*).

**Proposition 8**

If *μ* ∈ FI (*A*) and *μ*((*x* ^−^ → *y* ^−^)^−^) = *μ*(0), then *μ*(*x*) ≤ *μ*(*y*) for any *x*, *y* ∈ *A*. In particular, *μ*(*x*) ≤ *μ*(*y*) where *x* ^−^ ≤ *y* ^−^ for any *x*, *y* ∈ *A*.

**Proof**

Since *μ*(*y*) ≥ *μ*((*x* ^−^ → *y* ^−^)^−^)∧*μ*(*x*) = *μ*(0)∧*μ*(*x*) = *μ*(*x*), we have *μ*(*x*) ≤ *μ*(*y*).

As an immediate consequence of the proposition we have the following.

**Corollary 9 (see [15])**

If *μ* ∈ FI (*A*) and *y* ≤ *x* then *μ*(*x*) ≤ *μ*(*y*) for any *x*, *y* ∈ *A*.

**Proposition 10**

Let *μ* ∈ FI (*A*). Then for any *x* ∈ *A*, *μ*(*x*) = *μ*(0) if and only if *μ*(*x* ^−−^) = *μ*(0).

**Proof**

(⇒) Suppose that *μ*(*x*) = *μ*(0). By *μ* ∈ FI(*A*), we have

(3) μ(x−−)≥μ((x−→x−−−)−)∧μ(x)=μ((x−→x−)−)∧μ(x)=μ(0)∧μ(0)=μ(0).

Hence *μ*(*x* ^−−^) = *μ*(0).

(⇐) Assume that *μ*(*x* ^−−^) = *μ*(0). Since for any *x* ∈ *A*, *x* ≤ *x* ^−−^, by Corollary 9, *μ*(0) = *μ*(*x* ^−−^) ≤ *μ*(*x*), thus *μ*(*x*) = *μ*(0).

**Theorem 11**

A fuzzy set *μ* in *A* is a fuzzy ideal if and only if for all *x*, *y*, *z* ∈ *A*, *z* ^−^ → (*y* ^−^ → *x* ^−^) = 1 implies *μ*(*x*) ≥ *μ*(*y*)∧*μ*(*z*).

**Proof**

Assuming that *μ* is a fuzzy ideal of *A* and *z* ^−^ → (*y* ^−^ → *x* ^−^) = 1, then *z* ^−^ ≤ *y* ^−^ → *x* ^−^ = (*y* ^−^ → *x* ^−^)^−−^, and by Proposition 8 we have *μ*(*z*) ≤ *μ*((*y* ^−^ → *x* ^−^)^−^), so *μ*(*x*) ≥ *μ*((*y* ^−^ → *x* ^−^)^−^)∧*μ*(*y*) ≥ *μ*(*y*)∧*μ*(*z*).

Conversely, suppose that *z* ^−^ → (*y* ^−^ → *x* ^−^) = 1 implies *μ*(*x*) ≥ *μ*(*y*)∧*μ*(*z*) for all *x*, *y*, *z* ∈ *A*. Since *x* ^−^ → (*x* ^−^ → 0^−^) = *x* ^−^ → (*x* ^−^ → 1) = 1, so *μ*(*o*) ≥ *μ*(*x*)∧*μ*(*x*) = *μ*(*x*); (FI1) holds. By 1 = (*x* ^−^ → *y* ^−^) → (*x* ^−^ → *y* ^−^) = (*x* ^−^→*y* ^−^)^−−^ → (*x* ^−^ → *y* ^−^), then *μ*(*y*) ≥ *μ*((*x* ^−^→*y* ^−^)^−^)∧*μ*(*x*), (FI2) holds.

By induction and Theorem 11 we have the following.

**Corollary 12**

Let *μ* be a fuzzy set in *A*. *μ* is a fuzzy ideal if and only if, for any *x*, *y*_1_,…, *y*_*n*_ ∈ *A*, *y*_*n*_^−^ → (*y*_*n*−1_^−^ → (⋯→(*y*_1_^−^ → *x* ^−^)⋯)) = 1 implies *μ*(*x*) ≥ *μ*(*y*_1_)∧⋯∧*μ*(*y*_*n*_).

**Proposition 13**

Letting *A* be a *BL*-algebra and *x*, *a*, *b*, *a*_1_,…, *a*_*n*_, *b*_1_,…, *b*_*m*_ ∈ *A*, if *b*∗*a* → *x* = 1, *a*_1_∗ ⋯ ∗*a*_*n*_ → *a* = 1, *b*_1_∗ ⋯ ∗*b*_*m*_ → *b* = 1, then

(4) $$\begin{array}{c} b_{1}*\cdots *b_{m}*a_{1}*\cdots *a_{n}\to x=1. \end{array}$$

**Proof**

From *a*_1_∗ ⋯ ∗*a*_*n*_ → *a* = 1, *b*_1_∗ ⋯ ∗*b*_*m*_ → *b* = 1 it follows that *a*_1_∗ ⋯ ∗*a*_*n*_ ≤ *a*, *b*_1_∗ ⋯ ∗*b*_*m*_ ≤ *b*. Since the operation “∗” is isotone, so we have *a*_1_∗ ⋯ ∗*a*_*n*_∗*b*_1_∗ ⋯ ∗*b*_*m*_ ≤ *a*∗*b*. While *b*∗*a* → *x* = 1 implies *b*∗*a* ≤ *x*, hence

(5) $$\begin{array}{c} b_{1}*\cdots *b_{m}*a_{1}*\cdots *a_{n}\leq x, \end{array}$$

that is,

(6) $$\begin{array}{c} b_{1}*\cdots *b_{m}*a_{1}*\cdots *a_{n}⟶x=1. \end{array}$$

The proof is complete.

**Definition 14**

Let *f* be a fuzzy set in *A*. A fuzzy ideal *μ* is called to be generated by *f* if *f* ≤ *μ* and *f* ≤ *ν* implies *μ* ≤ *ν* for any fuzzy ideal *ν* in *A*. The fuzzy ideal generated by *f* will be denoted by (*f*].

It is worth noticing that this definition is well-defined because 1_*A*_ is a fuzzy ideal in *A*; for any *f* ∈ FI(*A*) we have *f* ≤ 1_*A*_ and the intersection of any family of fuzzy ideals in *A* is a fuzzy ideal in *A*.

**Example 15 (see [7])**

Let *A* = {0, *a*, *b*, 1}. Define ∗,→, ∨, and ∧ as follows:

(7) ∗0ab100000a00aab0abb10ab1,→0ab101111aa111b0a1110ab1,∨0ab100ab1aaab1bbbb111111,∧0ab100000a0aaab0abb10ab1.

Then (*A*; ∧, ∨, ∗, →, 0,1) is a *BL*-algebra. Define a fuzzy set *μ* in *A* by *μ*(*a*) = *μ*(*b*) = 0.5, *μ*(1) = 0, *μ*(0) = 0.8. It is easy to check that (*μ*](0) = 0.8, (*μ*](*a*) = (*μ*](*b*) = (*μ*](1) = 0.5.

**Theorem 16**

Let *μ* and *ν* be fuzzy sets in *A*.

- If *μ* is a fuzzy ideal in *A* then (*μ*] = *μ*.
- If *μ* ≤ *ν* then (*μ*]≤(*ν*].
- (1_*A*_] = 1_*A*_, (0_*A*_] = 0_*A*_.

**Proof**

Trivial.

**Theorem 17**

Let *f* be a fuzzy set in *A*. If a fuzzy set *μ* in *A* is defined as follows, for any *x* ∈ *A*,

(8) μ(x)=∨{f(a1)∧⋯∧f(an) ∣ a1−∗⋯∗an−  ⟶x−=1 for  some  a1,…,an∈A},

then *μ* = (*f*].

**Proof**

First, we prove *μ* is a fuzzy ideal in *A*. Suppose that *x* ^−^ → (*y* ^−^ → *z* ^−^) = 1 (i.e., *x* ^−^∗*y* ^−^ → *z* ^−^ = 1) for any *x*, *y*, *z* ∈ *A*. Given any arbitrary small *ɛ* > 0, there exists *a*_1_,…, *a*_*n*_; *b*_1_,…, *b*_*m*_ ∈ *A*, such that

(9) $$\begin{array}{c} a_{1}^{-}*\cdots *a_{n}^{-}⟶x^{-}=1,\\ b_{1}^{-}*\cdots *b_{m}^{-}⟶y^{-}=1,\\ \mu \left(x\right)-ɛ<f\left(a_{1}\right)\wedge \cdots \wedge f\left(a_{n}\right),\\ \mu \left(y\right)-ɛ<f\left(b_{1}\right)\wedge \cdots \wedge f\left(b_{m}\right). \end{array}$$

By Proposition 13 we get

(10) $$\begin{array}{c} b_{1}^{-}*\cdots *b_{m}^{-}*a_{1}^{-}*\cdots *a_{n}^{-}\to z^{-}=1. \end{array}$$

It follows that

(11) μ(z)≥f(bm)∧⋯∧f(b1)∧f(an)∧⋯∧f(a1)>(μ(x)−ɛ)∧(μ(y)−ɛ)=μ(x)∧μ(y)−ɛ.

Hence *μ*(*z*) ≥ *μ*(*x*)∧*μ*(*y*), and *μ* is a fuzzy ideal in *A* by Theorem 11.

Next, since *x* ^−^ → *x* ^−^ = 1 for any *x* ∈ *A*, it follows that *f*(*x*) ≤ *μ*(*x*), so *f* ≤ *μ*.

Finally, supposing that *ν* is any fuzzy ideal in *A* with *f* ≤ *ν*, then *f*(*x*) ≤ *ν*(*x*) for any *x* ∈ *A*,

(12) μ(x)=∨{f(a1)∧⋯∧f(an) ∣ a1−∗⋯∗an−⟶x−=1}≤∨{ν(a1)∧⋯∧ν(an) ∣ a1−∗⋯∗an−⟶x−=1}≤∨{ν(x)}=ν(x),

by Corollary 12, so *μ* ≤ *ν*.

From the above we prove *μ* = (*f*].

**Notation 1**

In the sequel we need the notion of fuzzy points. Let *a*_*s*_ be a fuzzy set in *A* as follows:

(13) $$\begin{array}{c} a_{s}\left(x\right)=\{\begin{array}{cc} s, & \text{if}\;\;x=a;\\ 0, & \text{if}\;\;x\neq a, \end{array} \end{array}$$

where *a* ∈ *A* and *s* ∈ [0,1], and then *a*_*s*_ is called a fuzzy point in *A* with value *s* at *a*.

**Proposition 18 (see [18])**

Let *A* be a *BL*-algebra. For any *x*, *y*, *z* ∈ *A* and *n*, *m* ∈ *N*, if *y* ^*n*^ → *x* = *z* ^*m*^ → *x* = 1, then there exists *p* ∈ *N* such that (*y*∨*z*)^*p*^ → *x* = 1.

**Theorem 19**

Let *μ* be a fuzzy ideal in *A*. If *s*, *t* ∈ [0,1] satisfies *s* ≥ *μ*(*a*), *t* ≥ *μ*(*b*), *s*∧*t* ≤ *μ*(*a*∧*b*) where *a*, *b* ∈ *A*, then

(14) $$\begin{array}{c} \left(\mu \vee a_{s}\right]\wedge \left(\mu \vee b_{t}\right]=\mu . \end{array}$$

**Proof**

It is obvious that *μ* ≤ (*μ*∨*a*_*s*_]∧(*μ*∨*b*_*t*_], so we just need to prove the converse inequality. Observe for all *x* ∈ *A*,

(15) $$\begin{array}{c} \left(\left(\mu \vee a_{s}\right]\wedge \left(\mu \vee b_{t}\right]\right)\left(x\right)=\left(\mu \vee a_{s}\right]\left(x\right)\wedge \left(\mu \vee b_{t}\right]\left(x\right). \end{array}$$

Suppose we are given any fixed *x* ∈ *A* and an arbitrary small *ɛ* > 0, it is sufficient to consider the following three cases.

*Case I. *There are *a*_1_, *a*_2_,…, *a*_*n*_ ∈ *A*∖{*a*} such that

- *a*_1_^−^∗ ⋯ ∗*a*_*n*_^−^ → *x* ^−^ = 1,
- (*μ*∨*a*_*s*_](*x*) − *ɛ* < (*μ*∨*a*_*s*_)(*a*_1_)∧⋯∧(*μ*∨*a*_*s*_)(*a*_*n*_).

Since (*μ*∨*a*_*s*_)(*a*_*i*_) = *μ*(*a*_*i*_)  (*i* = 1,…, *n*), we obtain

(16) $$\begin{array}{c} \left(\mu \vee a_{s}\right]\left(x\right)<\mu \left(a_{1}\right)\wedge \cdots \wedge \mu \left(a_{n}\right)+ɛ\leq \mu \left(x\right)+ɛ. \end{array}$$

Hence

(17) $$\begin{array}{c} \left(\left(\mu \vee a_{s}\right]\wedge \left(\mu \vee b_{t}\right]\right)\left(x\right)\leq \mu \left(x\right)+ɛ. \end{array}$$

*Case II.* There are *b*_1_, *b*_2_,…, *b*_*m*_ ∈ *A*∖{*b*} such that

- *b*_1_^−^∗ ⋯ ∗*b*_*m*_^−^ → *x* ^−^ = 1,
- (*μ*∨*b*_*t*_](*x*) − *ɛ* < (*μ*∨*b*_*t*_)(*b*_1_)∧⋯∧(*μ*∨*b*_*t*_)(*b*_*m*_).

By the way similar to Case I we also obtain

(18) $$\begin{array}{c} \left(\left(\mu \vee a_{s}\right]\wedge \left(\mu \vee b_{t}\right]\right)\left(x\right)\leq \mu \left(x\right)+ɛ. \end{array}$$

*Case III.* There are *a*_1_, *a*_2_,…, *a*_*n*_ ∈ *A*∖{*a*} and *l* ∈ *N* such that

- (*a* ^−^)^*l*^∗*a*_1_^−^∗ ⋯ ∗*a*_*n*_^−^ → *x* ^−^ = 1,
- (*μ*∨*a*_*s*_](*x*) − *ɛ* < (*μ*∨*a*_*s*_)(*a*_1_)∧⋯∧(*μ*∨*a*_*s*_)(*a*_*n*_)∧(*μ*∨*a*_*s*_)(*a*) = *μ*(*a*_1_∧⋯∧*μ*(*a*_*n*_)∧*s*.

Also there are *b*_1_, *b*_2_,…, *b*_*m*_ ∈ *A*∖{*b*} and *k* ∈ *N* such that

(iii) (*b* ^−^)^*k*^∗*b*_1_^−^∗ ⋯ ∗*b*_*m*_^−^ → *x* ^−^ = 1,
(iv) (*μ*∨*b*_*t*_](*x*) − *ɛ* < (*μ*∨*b*_*t*_)(*b*_1_)∧⋯∧(*μ*∨*b*_*t*_)(*b*_*m*_)∧(*μ*∨*b*_*t*_)(*b*) = *μ*(*b*_1_)∧⋯∧*μ*(*b*_*m*_)∧*t*.

Because *a*_1_^−^∗ ⋯ ∗*a*_*n*_^−^∗*b*_1_^−^∗ ⋯ ∗*b*_*m*_^−^ ≤ *a*_1_^−^∗ ⋯ ∗*a*_*n*_^−^, by Proposition 13 and (i) we get

(19) $$\begin{array}{c} {\left(a^{-}\right)}^{l}*a_{1}^{-}*\cdots *a_{n}^{-}*b_{1}^{-}*\cdots *b_{m}^{-}⟶x^{-}=1, \end{array}$$

that is,

(i′) (*a* ^−^)^*l*^ → (*a*_1_^−^∗ ⋯ ∗*a*_*n*_^−^∗*b*_1_^−^∗ ⋯ ∗*b*_*m*_^−^ → *x* ^−^) = 1.

By the similar argument and (iii) we can get

(iii′) (*b* ^−^)^*k*^ → (*a*_1_^−^∗ ⋯ ∗*a*_*n*_^−^∗*b*_1_^−^∗ ⋯ ∗*b*_*m*_^−^ → *x* ^−^) = 1.

By (i′), (iii′), and Proposition 18 there is a *p* ∈ *N* such that

(20) $$\begin{array}{c} {\left({\left(a\wedge b\right)}^{-}\right)}^{p}\to \left(a_{1}^{-}*\cdots *a_{n}^{-}*b_{1}^{-}*\cdots *b_{m}^{-}⟶x^{-}\right)=1. \end{array}$$

Thus

(21) $$\begin{array}{c} a_{1}^{-}*\cdots *a_{n}^{-}*b_{1}^{-}*\cdots *b_{m}^{-}*{\left({\left(a\wedge b\right)}^{-}\right)}^{p}⟶x^{-}=1, \end{array}$$

so we have

(22) μ(x)+ɛ ≥μ(a1)∧⋯∧μ(an)∧μ(b1)∧⋯μ(bm)∧μ(a∧b)+ɛ ≥μ(a1)∧⋯∧μ(an)∧μ(b1)∧⋯∧μ(bm)  ∧(s∧t)+ɛ =[μ(a1)∧⋯∧μ(an)∧s+ɛ]  ∧[μ(b1)∧⋯∧μ(bm)∧t+ɛ] ={(μ∨as)(a1)∧⋯∧(μ∨as)(an)∧(μ∨as)(a)+ɛ}  ∧{(μ∨bt)(b1)∧⋯∧(μ∨bt)(bm)∧(μ∨bt)(b)+ɛ} ≥(μ∨as](x)∧(μ∨bt](x).

This proves that (*μ*∨*a*_*s*_]∧(*μ*∨*b*_*t*_] ≤ *μ*. Thus (*μ*∨*a*_*s*_]∧(*μ*∨*b*_*t*_] = *μ*.

## 4. Fuzzy Prime Ideals and Fuzzy Irreducible Ideals

In this section we introduce the notions of fuzzy prime ideals and fuzzy irreducible ideals and investigate their properties. The emphasis is relation between fuzzy prime ideals and fuzzy irreducible ideals.

**Definition 20**

A nonconstant fuzzy ideal *μ* in *A* is called a fuzzy prime ideal in *A* if for any *x*, *y* ∈ *A*, *μ*(*x*∧*y*) ≤ *μ*(*x*)∨*μ*(*y*).

**Theorem 21**

A nonconstant fuzzy set *μ* in *A* is a fuzzy prime ideal in *A* if and only if *μ*_*t*_ = *∅* where *t* > *μ*(0); *μ*_*t*_ is a prime ideal of *A* where inf⁡{*μ*(*x*) | *x* ∈ *A*} < *t* ≤ *μ*(0); *μ*_*t*_ = *A* where 0 ≤ *t* ≤ inf⁡{*μ*(*x*) | *x* ∈ *A*}.

**Proof**

It is easy and omitted.

**Example 22**

Let *a*, *b* ∈ [0,1] and *a* < *b*. If *I* is a prime ideal of *A* and *J* is an proper ideal of *A* with *I* ⊂ *J*, then the function *f*_*IJ*_^*ab*^ : *A* → [0,1] is a fuzzy prime ideal in *A* where

(23) $$\begin{array}{c} f_{IJ}^{ab}\left(x\right)=\{\begin{array}{cc} b, & \text{if}\;\;x\in I,\\ a, & \text{if}\;\;x\in J-I,\\ 0, & \text{if}\;\;x\notin J. \end{array} \end{array}$$

As a special case of the above example we have the following.

**Example 23**

If *I* is a prime ideal of *A*, then the characteristic function *χ*_*I*_ of *I* is a fuzzy prime ideal in *A* where

(24) $$\begin{array}{c} \chi_{I}\left(x\right)=\{\begin{array}{cc} 1, & \text{if}\;\;x\in I,\\ 0, & \text{if}\;\;x\notin I. \end{array} \end{array}$$

**Theorem 24**

Letting *μ* be a fuzzy ideal in A, then *μ* is a fuzzy prime ideal in *A* if and only if *μ*_*μ*(0)_ is a prime ideal of *A*.

**Proof**

The “only if” part is easy. We now prove the part “if” as follows. Suppose that *μ*_*μ*(0)_ is a prime ideal of *A*. *μ*_*t*_ = *∅* where *t* > *μ*(0); *μ*_*μ*(0)_⊆*μ*_*t*_ ≠ *A* where inf⁡{*μ*(*x*) | *x* ∈ *A*} < *t* ≤ *μ*(0), so *μ*_*t*_ is a prime ideal of *A*; *μ*_*t*_ = *A* where 0 ≤ *t* ≤ inf⁡{*μ*(*x*) | *x* ∈ *A*}. Thus *μ* is a fuzzy prime ideal in *A* by Theorem 21.

**Theorem 25**

A nonconstant fuzzy ideal *μ* in *A* is a fuzzy prime ideal in *A* if and only if *μ*(*x*∧*y*) = *μ*(0) implies *μ*(*x*) = *μ*(0) or *μ*(*y*) = *μ*(0).

**Proof**

Suppose that *μ* is a fuzzy prime ideal in *A*; then *μ*_*μ*(0)_ is a prime ideal of *A* by Theorem 24. If *μ*(*x*∧*y*) = *μ*(0), then *x*∧*y* ∈ *μ*_*μ*(0)_, and so *x* ∈ *μ*_*μ*(0)_ or *y* ∈ *μ*_*μ*(0)_. Hence *μ*(*x*) = *μ*(0) or *μ*(*y*) = *μ*(0).

Conversely, suppose, for any *x*, *y* ∈ *A*, *μ*(*x*∧*y*) = *μ*(0) implies *μ*(*x*) = *μ*(0) or *μ*(*y*) = *μ*(0). That is, *x*∧*y* ∈ *μ*_*μ*(0)_ implies *x* ∈ *μ*_*μ*(0)_ or *y* ∈ *μ*_*μ*(0)_, and thus *μ*_*μ*(0)_ is a prime ideal of *A*. Therefore *μ* is a fuzzy prime ideal in *A* by Theorem 24.

*Note.* The above theorem shows that the definition on fuzzy prime ideals in this paper and one in [15] are equivalent.

The following corollary is easy and the proof is omitted.

**Corollary 26 (see [15])**

A nonconstant fuzzy ideal *μ* of *A* is a fuzzy prime ideal if and only if *μ*((*x* → *y*)^−^) = *μ*(0) or *μ*((*y* → *x*)^−^) = *μ*(0) for any *x*, *y* ∈ *A*.

We will call the next theorem as the extension theorem of fuzzy prime ideals.

**Theorem 27**

Let *μ* be a fuzzy prime ideal in *A*, *ν* be a nonconstant fuzzy ideal in *A*. If *μ* ≤ *ν* and *μ*(0) = *ν*(0), then *ν* is a fuzzy prime ideal.

**Proof**

Supposing that *μ* is a fuzzy prime ideal in *A*, then *μ*((*x* → *y*)^−^) = *μ*(0) or *μ*((*y* → *x*)^−^) = *μ*(0) for any *x*, *y* ∈ *A* by Corollary 26. If *μ*((*x* → *y*)^−^) = *μ*(0), by *μ* ≤ *ν* and *μ*(0) = *ν*(0), we have *ν*(0) = *μ*(0) = *μ*((*x* → *y*)^−^) ≤ *ν*((*x* → *y*)^−^), so *ν*((*x* → *y*)^−^) = *ν*(0). Likewise, if *μ*((*y* → *x*)^−^) = *μ*(0), then *ν*((*y* → *x*)^−^) = *ν*(0), so *ν* is a fuzzy prime ideal in *A*.

In what follows we introduce another notion—fuzzy irreducible ideals, and discuss relation between fuzzy prime ideals and fuzzy irreducible ideals.

**Definition 28**

A nonconstant fuzzy ideal *ω* in *A* is called a* fuzzy irreducible ideal* if, for any fuzzy ideals *μ* and *ν* in *A*, *μ*∧*ν* = *ω* implies *μ* = *ω* or *ν* = *ω*.

**Theorem 29**

Let *ω* be a nonconstant fuzzy ideal in *A*. If *ω* is a fuzzy irreducible ideal in *A* then *ω* is a fuzzy prime ideal in *A*.

**Proof**

Suppose *ω* is a fuzzy irreducible ideal in *A*. If *ω* is not a fuzzy prime ideal in *A*, then there are *a*, *b* ∈ *A* such that *ω*(*a*)∨*ω*(*b*) < *ω*(*a*∧*b*). Denote *s* = (1/2){[*ω*(*a*)∨*ω*(*b*)] + *ω*(*a*∧*b*)} and *μ* = (*ω*∨*a*_*s*_], *ν* = (*ω*∨*b*_*s*_]. Since *ω*(*a*)∨*ω*(*b*) < *s* < *ω*(*a*∧*b*), by Theorem 19 we have *μ*∧*ν* = *ω*, but *μ* ≠ *ω*, *ν* ≠ *ω*, a contradiction.

But the converse of the above theorem is not true.

**Example 30 (see [18])**

Let *A* = {0, *a*, *b*, 1}. Define ∗, →, ∨, and ∧ as follows:

(25) ∗0ab100000a0a0ab00bb10ab1,→0ab101111ab1b1baa1110ab1,∨0ab100ab1aaa11bb1b111111,∧0ab100000a0a0ab00bb10ab1.

Then (*A*; ∧, ∨, ∗, →, 0,1) is a *BL*-algebra. It is easy to check that *I*_1_ = {0, *a*}, *I*_2_ = {0, *b*} are prime ideals of *A*.

Define a fuzzy set *ω* in *A* by *ω*(0) = *ω*(*a*) = 1/2, *ω*(1) = *ω*(*b*) = 1/4, and then *ω* is a fuzzy prime ideal in *A*. Indeed, *ω*_*t*_ = *∅* where *t* > 1/2; *ω*_*t*_ = {0, *a*} where 1/4 < *t* ≤ 1/2; *ω*_*t*_ = *A* where 0 ≤ *t* ≤ 1/4. Let *μ* be a fuzzy ideal in *A* defined by *μ*(0) = 3/4, *μ*(*a*) = 3/4, *μ*(*b*) = *μ*(1) = 1/4. Let *ν* be a fuzzy ideal in *A* defined by *ν*(0) = *ν*(*a*) = 1/2, *ν*(*b*) = *ν*(1) = 3/8. It is easy to verify that *μ*∧*ν* = *ω* but *μ* ≠ *ω*, *ν* ≠ *ω*. Therefore *ω* is not a fuzzy irreducible ideal in *A*.

This example shows the converse of Theorem 29 is not true.

Letting *ξ* be a fuzzy set in *A* defined by *ξ*(0) = *ξ*(*a*) = 1, *ξ*(*b*) = *ξ*(1) = *t*(0 < *t* < 1), it is easy to check that *ξ* is a fuzzy irreducible ideal in *A*.

As is well-known, in ideal theory of *BL*-algebras, an ideal is prime if and only if it is irreducible [18]. But in the above we obtain an important fact:* in fuzzy ideal theory, any fuzzy irreducible ideal is a fuzzy prime ideal, but conversely a fuzzy prime ideal may not be a fuzzy irreducible ideal.*

Now we give general results.

**Lemma 31**

If *ω* is a fuzzy irreducible ideal in *A*, then *ω*(0) = 1.

**Proof**

Suppose *ω*(0) < 1. We just need to discuss the following two cases.

*Case I. *|*Im*⁡(*ω*)| = 2 where *Im*⁡(*ω*) = {*ω*(*x*) | *x* ∈ *A*}. Suppose *Im*⁡(*ω*) = {*ω*(0), *α*}. It is clear that 1 > *ω*(0) > *α*. Define fuzzy sets *μ* and *ν* as follows:

(26) $$\begin{array}{c} \mu \left(x\right)=\{\begin{array}{cc} 1, & \text{if}\;\;x\in \omega_{\omega (0)};\\ \alpha , & \text{if}\;\;x\in A-\omega_{\omega (0)}, \end{array}\\ \nu \left(x\right)=\{\begin{array}{cc} \omega \left(0\right), & \text{if}\;\;x\in \omega_{\omega (0)};\\ \frac{1}{2}\left(\omega \left(0\right)+\alpha \right), & \text{if}\;\;x\in A-\omega_{\omega (0)}. \end{array} \end{array}$$

Obviously, *μ* and *ν* are fuzzy ideals in *A*, and *μ*∧*ν* = *ω*, but *μ* ≠ *ω*, *ν* ≠ *ω*, a contradiction.

*Case II. *|*Im*⁡(*ω*)|>2 where *Im*⁡(*ω*) = {*ω*(*x*) | *x* ∈ *A*}. Then there is *t* ∈ (0,1) such that *ω*_*t*_⊃*ω*_*ω*(0)_, *ω*_*t*_ ≠ *ω*_*ω*(0)_, and (0, *t*)∩*Im*⁡(*ω*) ≠ *∅*. Define fuzzy sets *μ* and *ν* as follows:

(27) $$\begin{array}{c} \mu \left(x\right)=\{\begin{array}{cc} 1, & \text{if}\;\;\omega \left(x\right)>t;\\ \omega \left(x\right), & \text{if}\;\;\omega \left(x\right)\leq t, \end{array}\\ \nu \left(x\right)=\{\begin{array}{cc} \omega \left(x\right), & \text{if}\;\;\omega \left(x\right)>t;\\ t, & \text{if}\;\;\omega \left(x\right)\leq t. \end{array} \end{array}$$

Obviously, *μ* and *ν* are fuzzy ideals in *A*, and *μ*∧*ν* = *ω*, but *μ* ≠ *ω*, *ν* ≠ *ω*, a contradiction.

**Lemma 32**

Letting *ω* be a fuzzy prime ideal in *A* and |*Im*⁡(*ω*)|≥3, then *ω* is not a fuzzy irreducible ideal in *A*.

**Proof**

If *ω*(0) ≠ 1, then it follows from Lemma 31 that *ω* is not a fuzzy irreducible ideal in *A*.

If *ω*(0) = 1, then there are *s*_1_, *s*_2_ ∈ *Im*⁡(*ω*) such that 1 > *s*_1_ > *s*_2_. Define fuzzy sets *μ* and *ν* as follows: for all *x* ∈ *A*

(28) $$\begin{array}{c} \mu \left(x\right)=\{\begin{array}{cc} 1, & \text{if}\;\;\omega \left(x\right)\geq s_{1};\\ \omega \left(x\right), & \text{if}\;\;\omega \left(x\right)<s_{1}, \end{array}\\ \nu \left(x\right)=\{\begin{array}{cc} \omega \left(x\right), & \text{if}\;\;\omega \left(x\right)\geq s_{1};\\ s_{1}, & \text{if}\;\;\omega \left(x\right)<s_{1}. \end{array} \end{array}$$

Obviously, *μ* and *ν* are fuzzy ideals in *A*, and *μ*∧*ν* = *ω*, but *μ* ≠ *ω*, *ν* ≠ *ω*. Hence *ω* is not a fuzzy irreducible ideal in *A*.

**Lemma 33**

Letting *E* be a prime ideal of *A*, then the characteristic function *χ*_*E*_ is a fuzzy irreducible ideal in *A*.

**Proof**

Suppose *χ*_*E*_ is not a fuzzy irreducible ideal in *A*. Then there are fuzzy ideals *μ*, *ν* in *A* such that *μ*∧*ν* = *χ*_*E*_ but *μ* ≠ *χ*_*E*_, *ν* ≠ *χ*_*E*_. Thus for some *a*, *b* ∈ *A* − *E* such that *μ*(*a*) > 0, *ν*(*b*) > 0. Since *μ*(*a*) ≤ *μ*(*a*∧*b*) and *ν*(*b*) ≤ *ν*(*a*∧*b*), it follows that

(29) $$\begin{array}{c} 0<\mu \left(a\right)\wedge \nu \left(b\right)\leq \mu \left(a\wedge b\right)\wedge \nu \left(a\wedge b\right)=\chi_{E}\left(a\wedge b\right), \end{array}$$

and so *χ*_*E*_(*a*∧*b*) = 1; that is, *a*∧*b* ∈ *E* which contradicts *E* being a prime ideal of *A*. Therefore *χ*_*E*_ is a fuzzy irreducible ideal in *A*.

In this Lemma *χ*_*E*_ is also a fuzzy prime ideal in *A*. Hence this shows that under some special conditions, a fuzzy prime ideal in *A* may be a fuzzy irreducible ideal in *A*.

**Theorem 34**

Let *ω* be a fuzzy prime ideal in *A*. Then *ω* is a fuzzy irreducible ideal in *A* if and only if *ω*(0) = 1 and |*Im*⁡(*ω*)| = 2.

**Proof**

(⇒) Suppose that *ω* is a fuzzy irreducible ideal in *A*. By Lemma 31, *ω*(0) = 1. If |*Im*⁡(*ω*)|≥3, then *ω* is not a fuzzy irreducible ideal in *A* by Lemma 32, a contradiction. Hence |*Im*⁡(*ω*)| = 2.

(⇐) Suppose that *ω*(0) = 1 and |*Im*⁡(*ω*)| = 2. We can prove that *ω* is a fuzzy irreducible ideal in *A* by the argument in Lemma 33.

**Theorem 35**

Let *μ* be a nonconstant fuzzy ideal in *A* and let *a*_*λ*_ be a fuzzy point in *A* with *a*_*λ*_ ∉ *μ*. Then there is a fuzzy prime ideal *ν* in *A* satisfying *μ* ≤ *ν* and *a*_*λ*_ ∉ *ν*.

**Proof**

Since *a*_*λ*_ ∉ *μ*, we have *μ*(*a*) < *λ*. Denote

(30) $$\begin{array}{c} t=\frac{\mu \left(a\right)+\lambda }{2},\;\;s=\frac{1}{4}\left[3\mu \left(a\right)+\lambda \right]. \end{array}$$

Then *μ*_*t*_ is an ideal of *A* and *a* ∉ *μ*_*t*_, or *μ*_*t*_ = *∅*. We consider the following three cases.

*Case I.* If *a* ≠ 0 and *μ*_*t*_ ≠ *∅*, then by Lemma 4 there exists a prime ideal *P* of *A* such that *μ*_*t*_⊆*P* and *a* ∉ *P*. Define a fuzzy set *ν* in *A* as follows:

(31) $$\begin{array}{c} \nu \left(x\right)=\{\begin{array}{cc} 1, & \text{if}\;\;x\in P,\\ t, & \text{if}\;\;x\in A-P. \end{array} \end{array}$$

It is easy to see that *ν* is a fuzzy irreducible ideal in *A* with *μ* ≤ *ν* and *a*_*λ*_ ∉ *ν*.

*Case II.* If *a* ≠ 0 and *μ*_*t*_ = *∅*, then the ideal {0} does not contain *a*. By Lemma 4 there is a prime ideal *P* of *A* such that {0}⊆*P* and *a* ∉ *P*. Define a fuzzy set *ν* such that

(32) $$\begin{array}{c} \nu \left(x\right)=\{\begin{array}{cc} 1, & \text{if}\;\;x\in P,\\ t, & \text{if}\;\;x\in A-P. \end{array} \end{array}$$

It is easy to check that *ν* is a fuzzy irreducible ideal in *A* with *μ* ≤ *ν* and *a*_*λ*_ ∉ *ν*.

*Case III.* Suppose *a* = 0. We take any prime ideal *P* of *A*, and then 0 ∈ *P*. Define a fuzzy set *ν* such that

(33) $$\begin{array}{c} \nu \left(x\right)=\{\begin{array}{cc} t, & \text{if}\;\;x\in P,\\ s, & \text{if}\;\;x\in A-P. \end{array} \end{array}$$

It is easy to check that *ν* is a fuzzy prime ideal in *A* with *μ* ≤ *ν* and 0_*λ*_ ∉ *ν*.

**Corollary 36**

Let *μ* be a nonconstant fuzzy ideal of *A*. Then *μ* is the intersection of all fuzzy prime ideals in *A* containing *μ*.

**Proof**

It is immediate by Theorem 35.

This is the Krull-Stone representation theorem of fuzzy ideals in a *BL*-algebra.

## 5. Distributivity of Fuzzy Ideal Lattices

Before discussing the structure of fuzzy ideal lattices we first observe that for any *μ*_*α*_ ∈ FI(*A*), where *α* ∈ Λ, Λ is an index set (may be infinite), ∧{*μ*_*a*_ | *α* ∈ Λ} ∈ FI(*A*), but in general, ∨{*μ*_*a*_ | *α* ∈ Λ} ∉ FI(*A*).

**Example 37**

Let *A* be the *BL*-algebra defined in Example 30. It can check that *F*_1_ = {0, *a*}, *F*_2_ = {0, *b*} are ideals of *A*. Define two fuzzy set as follows:

(34) $$\begin{array}{c} f\left(x\right)=s,\;x\in \left\{0,a\right\};\;\;f\left(x\right)=t,\;x\in \left\{b,1\right\},\\ g\left(x\right)=s,\;x\in \left\{0,b\right\};\;\;g\left(x\right)=t,\;x\in \left\{a,1\right\}, \end{array}$$

where *s* > *t* ∈ [0,1]. we get the fuzzy set *f*∨*g*:

(35) $$\begin{array}{c} \left(f\vee g\right)\left(x\right)=s,\;\;x\in \left\{0,a,b\right\};\;\;\left(f\vee g\right)\left(1\right)=t. \end{array}$$

It is easy to see that *f*, *g* are fuzzy ideals in *A*, but *f*∨*g* is not a fuzzy ideal in *A* since (*f*∨*g*)_*s*_ = {0, *a*, *b*} is not an ideal of *A*.

Now we give the following definition: for any *μ*, *ν* ∈ FI(*A*), denote *μ*⊔*ν* = :(*μ*∨*ν*]; in general, for any *μ*_*λ*_ ∈ FI(*A*)(*λ* ∈ Λ ≠ *∅*),

(36) $$\begin{array}{c} ⊔\left\{\mu_{\lambda }\;|\;\lambda \in \Lambda \right\}=:\left(\vee \left\{\mu_{\lambda }\;|\;\lambda \in \Lambda \right\}\right]. \end{array}$$

**Theorem 38**

Letting *A* be a *BL*-algebra, then (FI(*A*); ⊔, ∧, 0_*A*_, 1_*A*_) is a complete distributive lattice where 0_*A*_ and 1_*A*_ are the least lower bound and the largest upper bound ofFI(*A*), respectively, and satisfies the following infinitely distributive law:

(DL) *μ*∧⊔{*ν*_*α*_ | *α* ∈ Λ} = ⊔{*μ*∧*ν*_*α*_ | *α* ∈ Λ}, for all *μ*, *ν*_*α*_ ∈ FI (*A*)(*α* ∈ Λ ≠ *∅*).

**Proof**

It is easy to verify that for any *μ*, *ν* ∈ FI(*A*),  *μ*⊔*ν* and *μ*∧*ν* are the supremum and the infimum in FI(*A*) of *μ* and *ν*, and 0_*A*_ ≤ *μ* ≤ 1_*A*_, so (FI(*A*); ⊔, ∧, 0_*A*_, 1_*A*_) is a bounded lattice. This lattice is obviously complete.

In order to check (DL) it suffices to prove, for any *ν*_*λ*_ ∈ FI(*A*)(*λ* ∈ Λ ≠ *∅*),

(37) $$\begin{array}{c} \mu \wedge ⊔\left\{\nu_{\alpha }\;|\;\alpha \in \Lambda \right\}\leq ⊔\left\{\mu \wedge \nu_{\alpha }\;|\;\alpha \in \Lambda \right\}. \end{array}$$

By Theorem 17, for any given *x* ∈ *A* and arbitrary small *ɛ* > 0, there exist *a*_1_, *a*_2_,…, *a*_*n*_ ∈ *A* such that

(38) an−∗⋯∗a1−⟶x−=1(or  an−⟶(⋯⟶(a1−⟶x−))=1),⊔{να ∣ α∈Λ}(x)−ɛ<[∨{να ∣ α∈Λ}(a1)]  ∧⋯∧[∨{να ∣ α∈Λ}(an)].

By the definition of ∨{*ν*_*α*_ | *α* ∈ Λ}(*a*_*i*_)  (*i* = 1,…, *n*), there are *α*_1_,…, *α*_*n*_ ∈ Λ such that

(39) ∨{να ∣ α∈Λ}(a1)−ɛ<να1(a1),         ⋮∨{να ∣ α∈Λ}(an)−ɛ<ναn(an),

and thus we have

(40) ⊔{να ∣ α∈Λ}(x)<{[∨{να ∣ α∈Λ}(a1)]∧⋯∧[∨{να ∣ α∈Λ}(an)]}+ɛ<{[να1(a1)+ɛ]∧⋯∧[ναn(an)+ɛ]}+ɛ=[να1(a1)∧⋯∧ναn(an)]+2ɛ,

so

(41) (μ∧⊔{να ∣ α∈Λ})(x)=μ(x)∧⊔{να ∣ α∈Λ}(x)≤[να1(a1)∧⋯∧ναn(an)+2ɛ]∧μ(x)≤{[να1(a1)∧μ(x)]∧⋯∧[ναn(an)∧μ(x)]}+2ɛ.

Denote

(42) bn=(an−1−⟶(⋯⟶(a1−⟶x−)⋯))− =(an−1−∗⋯∗a1−→x−)−,

(43) bn−1=(an−2−⟶(⋯⟶(a1−⟶(bn−⟶x−))⋯))−=(an−2−∗⋯∗a1−∗bn−→x−)−,⋮

(44) b2=(a1−⟶(b3−⟶(⋯⟶(bn−⟶x−)⋯)))−=(a1−∗b3−∗⋯∗bn−⟶x−)−,

(45) b1=(b2−⟶(b3−⟶(⋯⟶(bn−⟶x−)⋯)))−=(b2−∗b3−∗⋯∗bn−→x−)−,

and then we get

(46) bn−=an−1−⟶(⋯⟶(a1−⟶x−)⋯) =an−1−∗⋯∗a1−⟶x−,

(47) bn−1−=an−2−⟶(⋯⟶(a1−⟶(bn−⟶x−))⋯)=an−2−∗⋯∗a1−∗bn−⟶x−,⋮

(48) b2−=a1−⟶(b3−⟶(⋯⟶(bn−⟶x−)⋯))=a1−∗b3−∗⋯∗bn−→x−,

(49) b1−=b2−⟶(b3−⟶(⋯⟶(bn−⟶x−)⋯))=b2−∗b3−∗⋯∗bn−⟶x−.

Therefore

(50) b1−⟶(b2−⟶(b3−⟶⋯⟶(bn−⟶x−)⋯))=1,b2−⟶(a1−⟶(b3−⟶⋯⟶(bn−⟶x−)⋯))=1, ⋮bn−⟶(an−1−⟶(⋯⟶(a1−⟶x−)⋯))=1.

Obviously we have

(51) $$\begin{array}{c} x^{-}\leq b_{1}^{-},b_{2}^{-},\ldots ,b_{n}^{-}. \end{array}$$

Thus by Proposition 8 we obtain the following.

(i) *μ*(*x*) ≤ *μ*(*b*_1_), *μ*(*b*_2_),…, *μ*(*b*_*n*_).

Since

(52) a1−⟶b1−=a1−⟶(b2−⟶(b3−⟶⋯⟶(bn−⟶x−)⋯))=b2−⟶(a1−⟶(b3−⟶⋯⟶(bn−⟶x−)⋯))=1,

so *a*_1_^−^ ≤ *b*_1_^−^. By a similar way we may prove that *a*_2_^−^ ≤ *b*_2_^−^,…, *a*_*n*_^−^ ≤ *b*_*n*_^−^. Therefore

(53) $$\begin{array}{c} a_{1}^{-}\leq b_{1}^{-},\ldots ,a_{n}^{-}\leq b_{n}^{-}. \end{array}$$

By Proposition 8 it follows that

(ii) *ν*_*α*_1__(*a*_1_) ≤ *ν*_*α*_1__(*b*_1_),…, *ν*_*α*_*n*__(*a*_*n*_) ≤ *ν*_*α*_*n*__(*b*_*n*_).

By (i) and (ii) we have

(54) μ(x)∧να1(a1)≤μ(b1)∧να1(b1),      ⋮μ(x)∧ναn(an)≤μ(bn)∧ναn(bn),

and so

(55) (μ∧⊔{να ∣ α∈Λ})(x)<{[να1(a1)∧μ(x)]∧⋯∧[ναn(an)∧μ(x)]}+2ɛ<{[να1(b1)∧μ(b1)]∧⋯∧[ναn(bn)∧μ(bn)]}+2ɛ=[(να1∧μ)(b1)∧⋯∧(ναn∧μ)(bn)]+2ɛ.

It is obvious

(56) (μ∧να1)(b1)≤∨{μ∧να ∣ α∈Λ}(b1),     ⋮(μ∧ναn)(bn)≤∨{μ∧να ∣ α∈Λ}(bn),

and hence

(57) (μ∧⊔{να ∣ α∈Λ})(x)  ≤{[∨{μ∧να ∣ α∈Λ}(b1)]    ∧⋯∧[∨{μ∧να ∣ α∈Λ}(bn)]}+2ɛ  ≤{[⊔{μ∧να ∣ α∈Λ}(b1)]    ∧⋯∧[⊔{μ∧να ∣ α∈Λ}(bn)]}+2ɛ.

Observe that

(58) $$\begin{array}{c} b_{1}^{-}⟶\left(b_{2}^{-}⟶\left(b_{3}^{-}⟶\cdots ⟶\left(b_{n}^{-}⟶x^{-}\right)\cdots \right)\right)=1, \end{array}$$

and ⊔{*μ*∧*ν*_*α*_ | *α* ∈ Λ} is a fuzzy ideal of *A*, and by Corollary 12 we get

(59) [⊔{μ∧να ∣ α∈Λ}(b1)]∧⋯∧[⊔{μ∧να ∣ α∈Λ}(bn)]  ≤⊔{μ∧να ∣ α∈Λ}(x).

Therefore

(60) $$\begin{array}{c} \left(\mu \wedge ⊔\left\{\nu_{\alpha }\;|\;\alpha \in \Lambda \right\}\right)\left(x\right)\leq ⊔\left\{\mu \wedge \nu_{\alpha }\;|\;\alpha \in \Lambda \right\}\left(x\right)+2ɛ. \end{array}$$

Since *ɛ* is arbitrary small, we have

(61) $$\begin{array}{c} \mu \wedge ⊔\left\{\nu_{\alpha }\;|\;\alpha \in \Lambda \right\}\leq ⊔\left\{\mu \wedge \nu_{\alpha }\;|\;\alpha \in \Lambda \right\}. \end{array}$$

The proof is completed.

## 6. Fuzzy Gödel Ideals 

In this section, we introduce the notion of fuzzy Gödel ideals of *BL*-algebra and investigate some of their properties.

**Definition 39**

Let *μ* be a fuzzy ideal of *A*. *μ* is called a fuzzy Gödel ideal if *μ*((*x* ^−^ → (*x* ^−^)^2^)^−^) = *μ*(0) for all *x* ∈ *A*.

It is obvious that each fuzzy ideal in Gödel algebra is a fuzzy Gödel ideal. In Example 48, we will show that there exists fuzzy Gödel ideal in non-Gödel algebra.

**Theorem 40**

Let *μ* be a fuzzy subset of *A*. *μ* is a fuzzy Gödel ideal if and only if, for each *t* ∈ [0,1], *μ*_*t*_ is a Gödel ideal of *A* where *μ*_*t*_ ≠ *∅*.

Next theorem is called the extension theorem of fuzzy Gödel ideals.

**Theorem 41**

Let *μ* and *ν* be fuzzy ideal of *A* with *μ* ≤ *ν* and *μ*(0) = *ν*(0). If *μ* is a fuzzy Gödel ideal, then so is *ν*.

**Notation 2**

Let *μ* be a fuzzy ideal of *A*. Define a fuzzy set *χ*_*μ*_ in *A* by

(62) $$\begin{array}{c} \chi_{\mu }\left(x\right)=\{\begin{array}{cc} \mu \left(0\right), & \text{if}\;\mu \left(x\right)=\mu \left(0\right);\\ 0, & \text{if}\;\mu \left(x\right)\neq \mu \left(0\right). \end{array} \end{array}$$

Obviously, *χ*_*μ*_ is also a fuzzy ideal in *A*.

**Theorem 42**

A fuzzy subset *μ* of *A* is a fuzzy Gödel ideal if and only if *χ*_*μ*_ is a fuzzy Gödel ideal of *A*.

**Proof**

If *μ* is a fuzzy Gödel ideal of *A*, then by Theorem 40, for any *t* ∈ [0,1], *μ*_*t*_ is a Gödel ideal of *A*. In particular, *μ*_*μ*(0)_ = {*x* ∈ *A* | *μ*(*x*) = *μ*(0)} is a Gödel ideal of *A*. We notice for any *t* ∈ [0,1]

(63) $$\begin{array}{c} {\left(\chi_{\mu }\right)}_{t}=\{\begin{array}{cc} \emptyset , & \text{if}\;\mu \left(0\right)<t;\\ \mu_{\mu (0)}, & \text{if}\;0<t\leq \mu \left(0\right);\\ A, & \text{if}\;t=0. \end{array} \end{array}$$

This shows that, for any *t* ∈ [0,1], (*χ*_*μ*_)_*t*_ is a Gödel ideal of *A* where (*χ*_*μ*_)_*t*_ ≠ *∅*. By Theorem 40 *χ*_*μ*_ is a fuzzy Gödel ideal of *A*.

Conversely, suppose *χ*_*μ*_ is a fuzzy Gödel ideal of *A*. It is clear that *χ*_*μ*_ ≤ *μ* and *χ*_*μ*_(0) = *μ*(0). By Theorem 41, *μ* is a fuzzy Gödel ideal of *A*.

**Theorem 43**

Let *μ* be a fuzzy ideal of *A*. The following conditions are equivalent:

- *μ* is a fuzzy Gödel ideal,
- *μ*(((*x* ^−^)^2^ → *y* ^−^)^−^) = *μ*(0) implies *μ*((*x* ^−^ → *y* ^−^)^−^) = *μ*(0),
- *μ*(((*x* ^−^∗*y* ^−^) → *z* ^−^)^−^) = *μ*(0) implies *μ*(((*x* ^−^ → *y* ^−^)→(*x* ^−^ → *z* ^−^))^−^) = *μ*(0).

**Proof**

(i)⇒(ii) Suppose that *μ* is a fuzzy Gödel ideal and *μ*(((*x* ^−^)^2^ → *y* ^−^)^−^) = *μ*(0). Then we have

(64) μ((x−⟶y−)−) ≥μ((((x−)2⟶y−)−−⟶(x−⟶y−)−−)−)  ∧μ(((x−)2⟶y−)−) =μ((((x−)2⟶y−)⟶(x−→y−))−)∧μ(0) ≥μ((x−⟶(x−)2)−)∧μ(0) =μ(0)∧μ(0)=μ(0).

Hence *μ*((*x* ^−^ → *y* ^−^)^−^) = *μ*(0).

(ii)⇒(iii) Suppose that *μ*(((*x* ^−^∗*y* ^−^) → *z* ^−^)^−^) = *μ*(0). Since *y* ^−^ → *z* ^−^ ≤ (*x* ^−^ → *y* ^−^)→(*x* ^−^ → *z* ^−^), then we have

(65) (x−∗y−)⟶z−=x−⟶(y−→z−)≤x−⟶((x−→y−)⟶(x−→z−))=x−⟶((x−⟶((x−⟶y−)⟶z−)))=(x−)2⟶((x−⟶y−)⟶z−),

and so ((*x* ^−^)^2^ → ((*x* ^−^ → *y* ^−^) → *z* ^−^))^−^ ≤ ((*x* ^−^∗*y* ^−^) → *z* ^−^)^−^. Hence

(66) μ(((x−)2⟶((x−⟶y−)⟶z−))−)  ≥μ(((x−∗y−)⟶z−)−)=μ(0),

and *μ*(((*x* ^−^)^2^ → ((*x* ^−^ → *y* ^−^) → *z* ^−^))^−^) = *μ*(0). From (ii) we get that

(67) $$\begin{array}{c} \mu \left({\left(x^{-}⟶\left(\left(x^{-}⟶y^{-}\right)⟶z^{-}\right)\right)}^{-}\right)=\mu \left(0\right), \end{array}$$

and hence

(68) $$\begin{array}{c} \mu \left({\left(\left(x^{-}⟶y^{-}\right)⟶\left(x^{-}⟶z^{-}\right)\right)}^{-}\right)=\mu \left(0\right). \end{array}$$

(iii)⇒(i) Since *μ*(((*x* ^−^)^2^ → (*x* ^−^)^2^)^−^) = *μ*(0), by (iii) we have

(69) $$\begin{array}{c} \mu \left({\left(\left(x^{-}⟶x^{-}\right)\to \left(x^{-}⟶{\left(x^{-}\right)}^{2}\right)\right)}^{-}\right)=\mu \left(0\right), \end{array}$$

and hence

(70) μ((x−⟶(x−)2)−) =μ((1⟶(x−⟶(x−)2))−) =μ(((x−⟶x−)⟶(x−⟶(x−)2))−)=μ(0),

and thus *μ* is a fuzzy Gödel ideal.

**Theorem 44**

Let *μ* be a fuzzy ideal. *μ* is a fuzzy Gödel ideal if and only if *μ*((*x* ^−^ → (*y* ^−^ → *z* ^−^))^−^) = *μ*(0) and *μ*((*x* ^−^ → *y* ^−^)^−^) = *μ*(0) imply *μ*((*x* ^−^ → *z* ^−^)^−^) = *μ*(0).

**Proof**

Suppose that *μ* is a fuzzy Gödel ideal. Let

(71) $$\begin{array}{c} \mu \left({\left(x^{-}⟶\left(y^{-}⟶z^{-}\right)\right)}^{-}\right)=\mu \left(0\right),\\ \mu \left({\left(x^{-}⟶y^{-}\right)}^{-}\right)=\mu \left(0\right). \end{array}$$

By Theorem 43(iii) we have

(72) $$\begin{array}{c} \mu \left({\left(\left(x^{-}⟶y^{-}\right)⟶\left(x^{-}⟶z^{-}\right)\right)}^{-}\right)=\mu \left(0\right). \end{array}$$

Then it follows that

(73) μ((x−⟶z−)−)  ≥μ(((x−⟶y−)−−⟶(x−⟶z−)−−)−)   ∧μ((x−⟶y−)−)  =μ(((x−⟶y−)⟶(x−⟶z−))−)   ∧μ((x−⟶y−)−)  =μ(0)∧μ(0)=μ(0),

and hence *μ*((*x* ^−^ → *z* ^−^)^−^) = *μ*(0).

Conversely, since *μ*((*x* ^−^ → (*x* ^−^ → (*x* ^−^)^2^))^−^) = *μ*(0) and *μ*((*x* ^−^ → *x* ^−^)^−^) = *μ*(0), then by the assumption we have *μ*((*x* ^−^ → (*x* ^−^)^2^)^−^) = *μ*(0), so *μ* is a fuzzy Gödel ideal.

**Theorem 45**

Let *μ* be a fuzzy ideal. Then *μ* is a fuzzy Gödel ideal if and only if the following condition holds:

(∗) *μ*(*x* ^−^ → ((*y* ^−^)^2^ → *z* ^−^)^−^) = *μ*(0) and *μ*(*x*) = *μ*(0) imply *μ*((*y* ^−^ → *z* ^−^)^−^) = *μ*(0) for any *x*, *y*, *z* ∈ *A*.

**Proof**

Suppose that *μ* is a fuzzy Gödel ideal of *A*. Letting *μ*((*x* ^−^ → ((*y* ^−^)^2^ → *z* ^−^))^−^) = *μ*(0) and *μ*(*x*) = *μ*(0), then

(74) μ(((y−)2⟶z−)−)  ≥μ((x−⟶((y−)2⟶z−)−−)−)∧μ(x)  =μ(x−⟶((y−)2⟶z−)−)∧μ(x)  =μ(0)∧μ(0)=μ(0),

and hence *μ*(((*y* ^−^)^2^ → *z* ^−^)^−^) = *μ*(0). Since *μ* is a fuzzy Gödel ideal, by Theorem 43(ii) we have *μ*((*y* ^−^ → *z* ^−^)^−^) = *μ*(0). (∗) holds.

Conversely, suppose that (∗) is true. Let *μ*((*x* ^−^ → (*y* ^−^ → *z* ^−^))^−^) = *μ*(0) and *μ*((*x* ^−^ → *y* ^−^)^−^) = *μ*(0). Since

(75) $$\begin{array}{c} {\left(x^{-}\right)}^{2}*\left(x^{-}⟶\left(y^{-}⟶z^{-}\right)\right)*\left(x^{-}⟶y^{-}\right)\leq z^{-}, \end{array}$$

then we have

(76) (x−⟶(y−⟶z−))∗(x−⟶y−)≤(x−)2⟶z−,x−⟶(y−⟶z−)≤(x−⟶y−)⟶((x−)2⟶z−),((x−⟶y−)⟶((x−)2⟶z−))−  ≤(x−⟶(y−⟶z−))−,μ((x−⟶(y−⟶z−))−) ≤μ(((x−⟶y−)⟶((x−)2⟶z−))−).

Since *μ*((*x* ^−^ → (*y* ^−^ → *z* ^−^))^−^) = *μ*(0), we get that

(77) $$\begin{array}{c} \mu \left({\left(\left(x^{-}⟶y^{-}\right)⟶\left({\left(x^{-}\right)}^{2}⟶z^{-}\right)\right)}^{-}\right)=\mu \left(0\right),\\ \mu \left({\left({\left(x^{-}⟶y^{-}\right)}^{--}⟶\left({\left(x^{-}\right)}^{2}⟶z^{-}\right)\right)}^{-}\right)=\mu \left(0\right). \end{array}$$

By (∗) and *μ*((*x* ^−^ → *y* ^−^)^−^) = *μ*(0) we get *μ*((*x* ^−^ → *z* ^−^)^−^) = *μ*(0). Thus *μ* is a fuzzy Gödel ideal by Theorem 44.

Zhang et al. [15]. introduced the notion of fuzzy Boolean ideals in *BL*-algebra and proved that fuzzy Boolean ideals are equivalent to fuzzy implicative ideals. In the following, we investigate the relation between fuzzy Boolean ideals and fuzzy Gödel ideals.

**Definition 46 (see [15])**

A fuzzy ideal *μ* in *A* is called a fuzzy Boolean ideal if *μ*(*x*∧*x* ^−^) = *μ*(0) for all *x* ∈ *A*.

**Theorem 47**

Each fuzzy Boolean ideal in *A* is a fuzzy Gödel ideal in *A*.

**Proof**

Suppose that *μ* is a fuzzy Boolean ideal in *A* and *μ*(((*x* ^−^)^2^ → *y* ^−^)^−^) = *μ*(0). Then

(78) μ((x−⟶y−)−) ≥μ(x∧x−)∧μ(((x∧x−)−⟶(x−⟶y−)−−)−) =μ(0)∧μ(((x−∨x−−)⟶(x−⟶y−))−) =μ(((x−∨x−−)∗x−⟶y−)−) ≥μ(((x−)2⟶y−)−)=μ(0),

and so *μ*((*x* ^−^ → *y* ^−^)^−^) = *μ*(0). By Theorem 43(ii) it follows that *μ* is a fuzzy Gödel ideal in *A*.

But the converse of the above theorem is not true.

**Example 48 (see [15])**

Let *A* = {0, *a*, *b*, *c*, *d*, 1}. Define ∗, →, ∨, and ∧ as follows:

(79) ∗0abcd10000000a0dc0dab0cbc0bc00c00cd0d00dd10abcd1,→0abcd10111111ac1bba1bda1adaca111a1db1bb1110abcd1,∨0abcd100abcd1aaa1aa1bb1bb01ccabca1dd11ad111abcd1,∧0abcd10000000a0accdab0cbc0bc0ccc0cd0d00dd10abcd1.

Then (*A*; ∧, ∨, ∗, →, 0,1) is a *BL*-algebra. Define a fuzzy set *μ* in *A* by *μ*(*a*) = *μ*(*b*) = *μ*(*c*) = *μ*(1) = 0.5, *μ*(0) = *μ*(*d*) = 0.8; one can easily check that *μ* is a fuzzy Gödel ideal in *A*, but it is not a fuzzy Boolean ideal, since *μ*(*a*∧*a* ^−^) = *μ*(*a*∧*c*) = *μ*(*c*) = 0.5 ≠ 0.8 = *μ*(0).

## 7. Conclusion

Study of fuzzy ideal theory in *BL*-algebras is technically more difficult, so far little research literature. Zhang et al. [15] initiated research in this area. In this paper we investigate further important properties of fuzzy ideals in *BL*-algebras. The notions of fuzzy prime ideals, fuzzy irreducible ideals, and fuzzy Gödel ideals are introduced and studied. We give a procedure to generate a fuzzy ideal by a fuzzy set. Using this result we prove that any fuzzy irreducible ideal is a fuzzy prime ideal and meanwhile we give an example to show that a fuzzy prime ideal may not be a fuzzy irreducible ideal; we also give the Krull-Stone representation theorem of fuzzy ideals in *BL*-algebras. Furthermore we prove that the set of all fuzzy ideals forms a complete distributive lattice. In addition, we prove that any fuzzy Boolean ideal in *BL*-algebras is a fuzzy Gödel ideal, but the converse is not true.

In our opinion, the future study of fuzzy ideals in *BL*-algebras should be related to (1) several special types of fuzzy ideals; (2) decomposition properties of fuzzy ideals. Our obtained results can be applied in information science, engineering, computer science, and medical diagnosis.
